# Mitigation of Dextran-Sodium-Sulfate-Induced Colitis in Mice through Oral Administration of Microbiome-Derived Inosine and Its Underlying Mechanisms

**DOI:** 10.3390/ijms241813852

**Published:** 2023-09-08

**Authors:** Weiling Guo, Xin Tang, Qiuxiang Zhang, Jianxin Zhao, Bingyong Mao, Hao Zhang, Shumao Cui

**Affiliations:** 1 State Key Laboratory of Food Science and Resources, Jiangnan University, Wuxi 214122, China; 7200112062@stu.jiangnan.edu.cn (W.G.); xintang@jiangnan.edu.cn (X.T.); zhangqx@jiangnan.edu.cn (Q.Z.); zhaojianxin@jiangnan.edu.cn (J.Z.); zhanghao61@jiangnan.edu.cn (H.Z.); cuishumao@jiangnan.edu.cn (S.C.); 2 School of Food Science and Technology, Jiangnan University, Wuxi 214122, China; 3 National Engineering Research Center for Functional Food, Jiangnan University, Wuxi 214122, China

**Keywords:** inosine, intestinal barrier, inflammatory responses, oxidative stress, gut microbiota

## Abstract

Background: Colonic and serum inosine are significantly reduced in patients with inflammatory bowel disease (IBD). Methods: This study aimed to explore whether microbiome-derived inosine alleviates colitis and its underlying mechanisms. Results: An inosine intervention effectively improved the clinical signs in colitis mice, suppressed inflammatory cytokines (tumor necrosis factor-alpha (TNF-α), interleukin-6 (IL-6), and IL-1β) by regulating the nuclear factor-kappa B (NF-κB) pathway, and elevated the activities of anti-oxidative enzymes (including superoxide dismutase (SOD) and glutathione peroxidase (GSH-Px)) by regulating the nuclear factor erythroid-2 related factor 2 (Nrf2) pathway. Additionally, the inosine intervention significantly elevated the expression of tight junction proteins (ZO-1, occudin, and claudin-1) in mice with colitis. High-throughput sequencing revealed that the inosine intervention also prevented gut microbiota disorder by increasing the abundance of beneficial bacteria (*Lachnospiraceae NK4A136 group*, *Romboutsia*, *Marvinbryantia*, *Clostridium sensu stricto* 1, and *Bifidobacterium*) and reducing the abundance of harmful bacteria (*Pseudomonas*, *Acinetobacter*, and *Tyzzerella*) in mice with colitis. Conclusions: Inosine played a significant role in mitigating colitis-related intestinal barrier injury and could potentially be used for therapy in clinical practice.

## 1. Introduction

Inflammatory bowel disease (IBD), a collective term for ulcerative colitis (UC) and Crohn’s disease (CD), is a category of idiopathic and chronic recurrent diseases. The prevalence of UC, in particular, has increased globally during the past few decades due to the long-term consumption of processed food, the abuse of antibiotics, unhealthy lifestyles, and environmental pollution [1]. For example, more than 3 million adults have been diagnosed as IBD patients in the USA, which may be partly due to the long-term consumption of alcohol and induced intestinal barrier injury [2,3]. Colitis is characterized by increases in the intestinal barrier permeability, abnormal inflammatory responses, and oxidative stress; the loss of tight junction proteins; and imbalances in the gut microbiota [4]. Increases in the permeability of the intestinal barrier promote the occurrence and development of some diseases associated with the inflammatory response because some harmful substances can enter the blood [5]. At present, some drugs are used to suppress colitis-related inflammation and the immune response to alleviate symptoms, such as tumor necrosis factor (TNF-α) therapy, corticosteroids, aminosalicylates, and antibiotics. However, the long-term consumption of these drugs may cause a range of unfavorable side effects and limited efficacy, such as high blood pressure, headaches, diabetes, liver injury, and nausea [6]. Thus, there is an urgent need to develop an effective and safe way of preventing and ameliorating IBD.

The gut microbiota plays a vital role in the severity and process of IBD. It can form a barrier on the mucosa, change the level of intestinal permeability, and strengthen the defense of the mucosal epithelium [7]. In IBD patients, the gut microbiota composition is altered [8]. The abundances of *Bifidobacterium* and *Lactobacillus* were dramatically reduced in IBD patients [9], while the relative abundances of harmful bacteria (including *Escherichia*_*Shigella*, *Parabacteroides*, *Romboutsia*, and *Turicibacter*) were significantly increased. A metabolomics analysis exhibited that some specific metabolites of gut microbes may be strongly associated with alterations in the gut microbiota [10]. For example, short-chain fatty acids (SCFAs) suppressed the production of inflammatory cytokines and the growth of harmful bacteria in the intestinal tract [11]. Li et al. found that the concentrations of colonic and serum inosine were significantly reduced in colitis mice compared with those in healthy mice [12]. In addition, the oral administration of inosine dramatically suppressed the production of inflammatory cytokines (TNF-α, interleukin (IL)-1β, and IL-6) and enhanced the activity of antioxidant enzymes in LPS-treated mice [13]. Mager et al. proved that microbiome-derived inosine enhanced the efficacy of checkpoint inhibitor immunotherapy in colorectal cancer by regulating the host’s immune system [14]. The above evidence indicates that inosine has the ability to inhibit the intestinal inflammatory response. However, the effect of inosine on the pathogenesis of colitis remains unknown.

In this study, we explored the influences of oral inosine intervention on inflammatory responses, oxidative stress, tight junction proteins, and gut microbiota in a colitis model. In addition, the transcriptional levels of the genes associated with inflammatory responses and oxidative stress were detected via RT-qPCR. A correlation analysis was applied to reveal the association between the gut microbial genus and some colitis-related indices. These results will offer new insights to understand the mechanisms of inosine in the treatment of colitis.

## 2. Results

### 2.1. Inosine Alleviated DSS-Induced Body Weight Loss, DAI Score, and Colon Shortening in Mice

**Figure 1:**
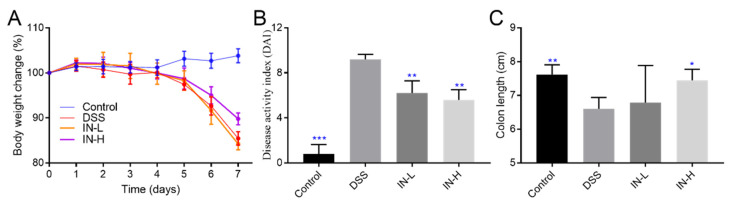
The role of microbiome-derived inosine in protecting the phenotypic index. Body weight change (**A**), DAI (**B**), and the colon length (**C**). * *p* < 0.05, ** *p* < 0.01, and *** *p* < 0.001 compared with the DSS group.

At the beginning of the DSS treatment, there were no obvious differences in body weight among all the groups (Figure 1). After 1 week of DSS treatment, the body weights of the mice in the DSS group were dramatically reduced to approximately 85.44% of their initial body weights compared to the healthy mice (*p* < 0.01). The inosine intervention effectively suppressed the DSS-induced body weight loss, especially in the IN-H group (which presented with approximately 89.78% of their initial body weights). The DAI score was extensively applied to evaluate the severity and development of colitis. Compared with the mice in the NC group, the DSS treatment dramatically elevated the DAI score (*p* < 0.001). Interestingly, the inosine interventions at 100 mg/kg and 800 mg/kg significantly suppressed the increases in the DAI scores (*p* < 0.01). There was a remarkable decrease in the colon length after the DSS treatment (*p* < 0.01). A high dose of inosine intervention remarkably reduced the effects on the DSS treatment (*p* < 0.01). These results exhibit that inosine may have the potential to improve colitis.

### 2.2. Inosine Alleviated DSS-Induced Inflammatory Cytokines in Mice

**Figure 2:**
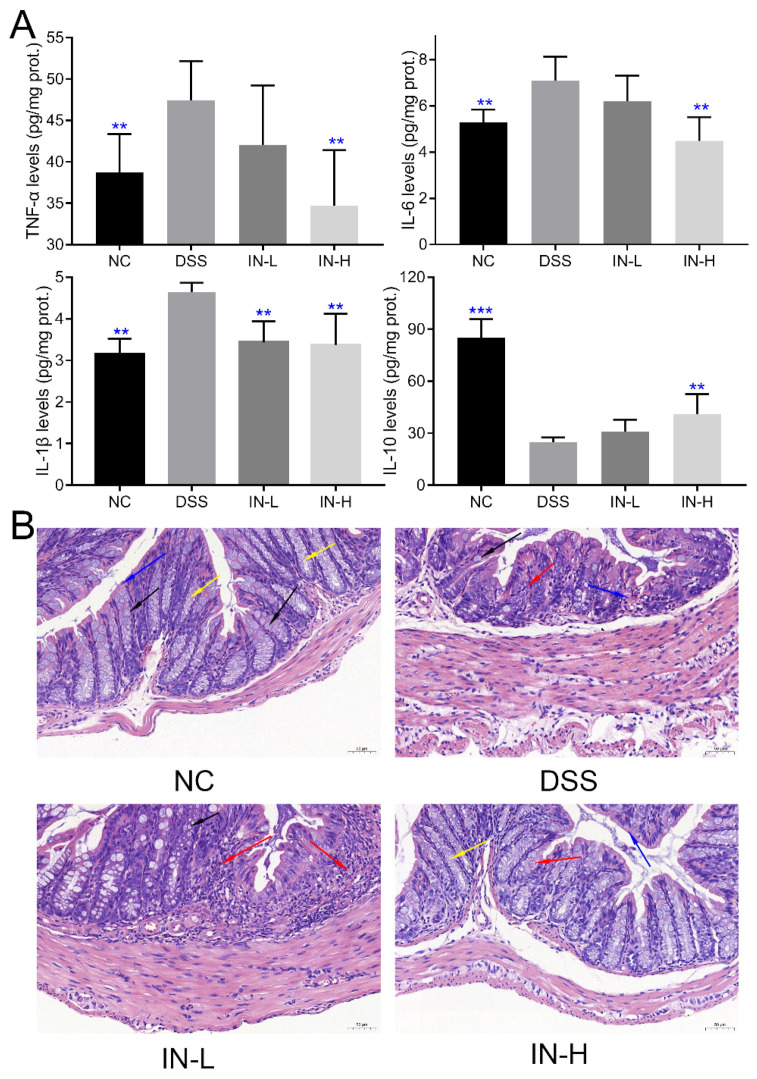
Effects of microbiome-derived inosine intervention on colonic inflammatory cytokines (**A**) and tissue damage (**B**) in DSS-treated mice. Inflammatory cell infiltration (red arrow), goblet cells (black arrow), mucosal epithelium (blue arrow), and crypt (yellow arrow). Scale bars, 50 μm. ** *p* < 0.01 and *** *p* < 0.001 compared with the DSS group.

As presented in Figure 2A, the colonic levels of TNF-α, IL-6, and IL-1β of the mice in the DSS group were notably elevated compared to those of the healthy mice in the NC group (*p* < 0.01), and a high dose of inosine intervention suppressed the DSS-induced elevation in the colonic levels of TNF-α, IL-6, and IL-1β (*p* < 0.01). However, the inosine intervention at 100 mg/kg only significantly reduced the colonic level of IL-1β in mice with colitis (*p* < 0.01). Additionally, the DSS treatment dramatically decreased the colonic level of IL-10 in the colitis mice compared to that of the mice in the NC group (*p* < 0.01). Interestingly, a high dose of inosine intervention significantly elevated the colonic level of IL-10 in mice with colitis (*p* < 0.01), while there was no significant difference in the colonic level of IL-10 in mice between the DSS and IN-L groups (*p* > 0.05). These results indicate that the inosine intervention improved the DSS-induced colitis in a dose-dependent manner.

To deeply explore the effect of inosine on the severity of inflammation and colon morphology of DSS-treated mice, the structure of the colon was observed after hematoxylin-eosin (H&E) staining. As presented in Figure 2B and Figure S1, the DSS treatment dramatically impaired the structure of the colon, including localized tissue ulcers, inflammatory cell infiltration, goblet cell depletion, mucosal epithelium and intestinal gland structure damage, crypt shortening, and absent mucosa. Notably, the supplementation of inosine could alleviate the severity of colon tissue damage to a certain extent, especially in the IN-H group. These results further confirm that the inosine intervention ameliorated the inflammation of the colon.

### 2.3. Inosine Elevated the Colonic Antioxidant Abilities in Mice with Colitis

**Figure 3:**
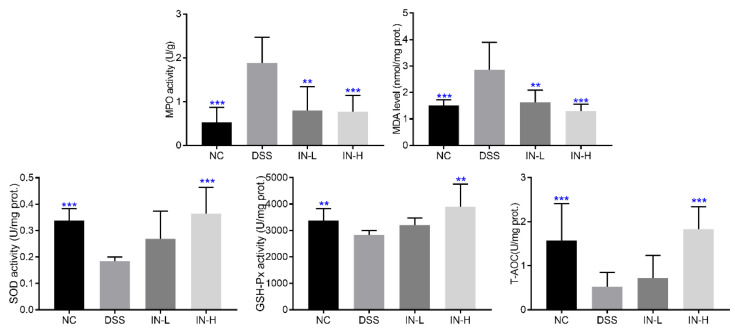
Effects of microbiome-derived inosine intervention on the oxidative stress in DSS-treated mice. ** *p* < 0.01 and *** *p* < 0.001 compared with the DSS group.

To assess the influence of inosine intervention on the colonic oxidative stress in DSS-treated mice, the colonic levels of MPO and MDA and the activities of SOD, GSH-Px, and T-AOC were determined (Figure 3). The DSS-treated mice displayed high colonic levels of MPO and MDA when compared with those of the healthy mice (*p* < 0.001). After an inosine intervention for two weeks, the colonic levels of MPO and MDA were dramatically reduced, especially in the IN-H group (*p* < 0.001). Moreover, the activities of SOD, GSH-Px, and T-AOC of the mice in the DSS group were significantly lower than those of the mice in the NC group (*p* < 0.01). However, a high dose of inosine intervention significantly elevated the activities of SOD, GSH-Px, and T-AOC in mice with colitis (*p* < 0.01). These results indicate that the inosine intervention ameliorated the capability of mice with colitis to suppress oxidative stress.

### 2.4. Inosine Prevented DSS-Induced Loss of Tight Junctional Proteins in Mice

**Figure 4:**
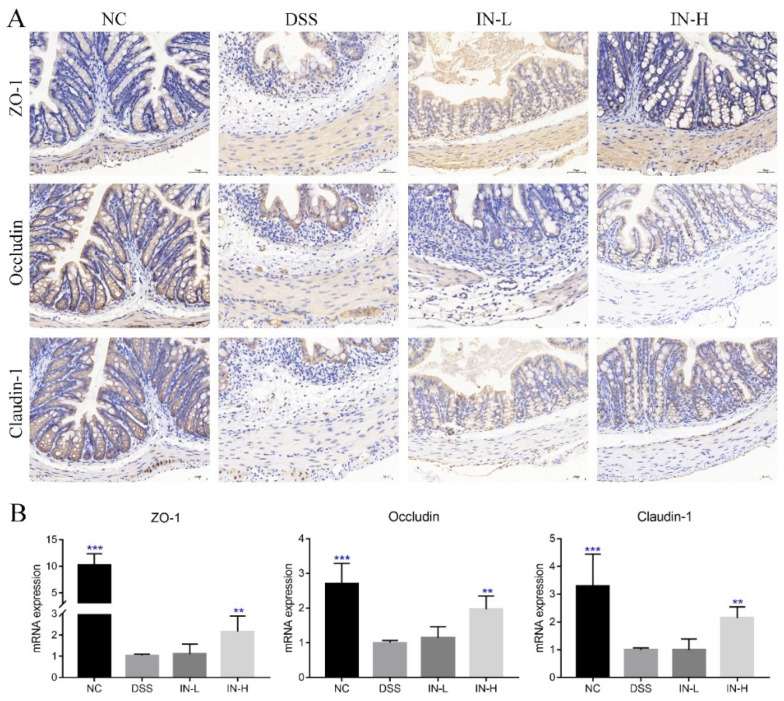
Microbiome-derived inosine preserved the intestinal barrier functions in DSS-treated mice. ZO-1, occludin, and claudin-1 in colon tissue were determined via immunohistochemical analysis (**A**). The transcription levels of ZO-1, occludin, and claudin-1 were detected via RT-qPCR (**B**). Scale bars, 50 μm. ** *p* < 0.01 and *** *p* < 0.001 compared with the DSS group.

As shown in Figure 4A, the immunohistochemical analysis revealed that the DSS treatment caused obvious decreases in the expressions of ZO-1, occludin, and claudin-1 in colon sections, which may promote the entry of harmful substances and microorganisms into the blood. Interestingly, the staining of these tight junction proteins of colonic tissues were stronger after the inosine intervention for two weeks, especially in the IN-H group. Moreover, the RT-PCR analysis further confirmed that the inosine intervention could up-regulate the transcriptional levels of ZO-1, occludin, and claudin-1 in mice with colitis, while a low dose of inosine intervention had little impact on their expression levels (Figure 4B).

### 2.5. Effects of Inosine on the Concentrations of Cecal SCFAs

The concentrations of cecal SCFAs in the cecal contents of the mice in the four groups were determined, including acetic, propanoic, isobutyric, butyric, valeric, and isovaleric acids. As presented in Table 1, the concentrations of cecal acetic, isobutyric, butyric, and isovaleric acids were significantly decreased in the DSS-treated mice compared with those in the healthy mice (*p* < 0.05), while the concentrations of cecal propanoic and valeric acids in the DSS-treated mice showed no significant differences compared with those of the mice in the NC group (*p* > 0.05). After two weeks of the inosine intervention, the concentration of cecal isovaleric acid was dramatically increased in mice with colitis (*p* < 0.05). Notably, there were no significant differences in the concentrations of cecal acetic, propanoic, isobutyric, butyric, and valeric acids in the mice among the DSS, IN-L, and IN-H groups (*p* > 0.05).

### 2.6. Inosine Regulated the Transcription Levels of Genes Related to Inflammation and Oxidative Stress in Mice with Colitis

**Figure 5:**
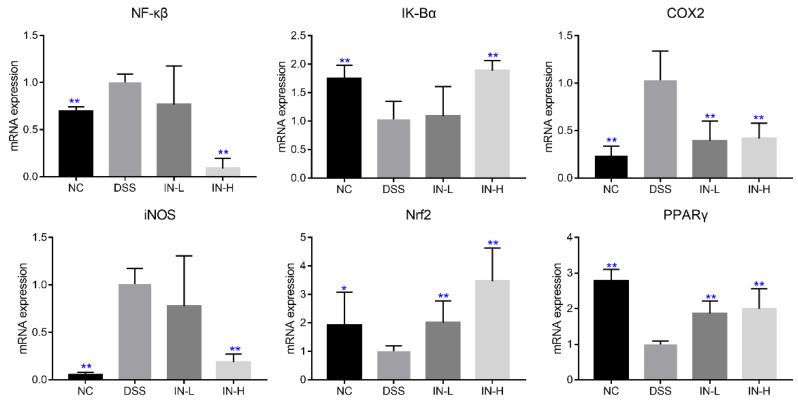
Effects of microbiome-derived inosine intervention on the mRNA transcription levels of genes related to inflammation and oxidative stress in DSS-treated mice. * *p* < 0.05 and ** *p* < 0.01 compared with the DSS group.

The mRNA transcription levels of genes related to inflammation and oxidative stress were detected via RT-PCR, including NF-κβ, IK-Bα, COX2, iNOS, Nrf2, and PPARγ. As presented in Figure 5, the transcription levels of NF-κβ, COX2, and iNOS were significantly up-regulated in the DSS group compared to those in the NC group, but the transcription levels of IK-Bα, Nrf2, and PPARγ were dramatically down-regulated (*p* < 0.05). Nevertheless, a high dose of inosine intervention could significantly recover the changes induced by DSS treatment (*p* < 0.01), but a low dose of inosine intervention only significantly suppressed the transcription level of COX2 in mice with colitis, and up-regulated the transcription levels of Nrf2 and PPARγ (*p* < 0.05).

### 2.7. Inosine Prevented DSS-Induced Gut Microbiota Disorder

**Figure 6:**
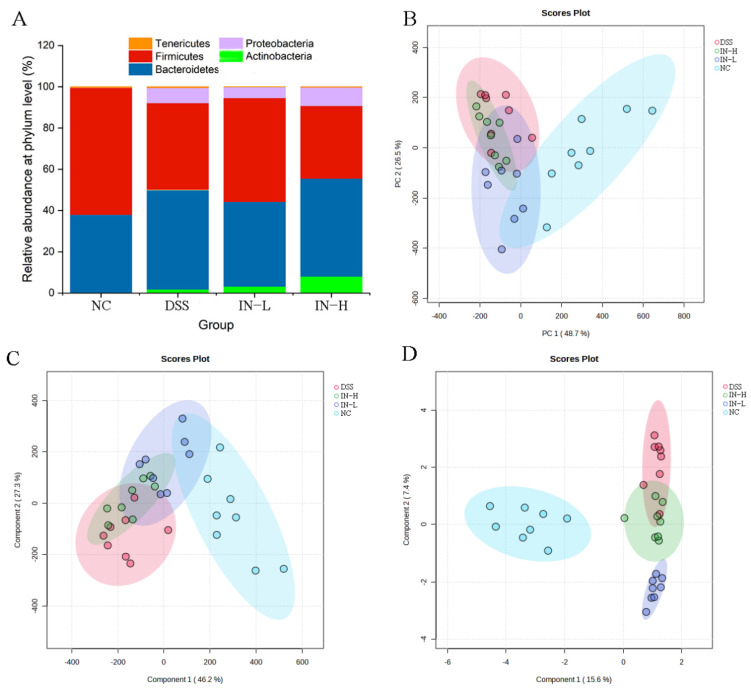
The regulative role of inosine in the gut microbiota composition. Relative abundance at the phylum level (**A**). PCA (**B**), PLS-DA (**C**), and SPLS-DA (**D**) analyses based on the genus level.

As presented in Figure 6A, Actinobacteria, Bacteroidetes, Firmicutes, Proteobacteria, and Tenericutes dominated the gut microbiota at the phylum level. The inosine intervention in particular increased the relative abundance of Firmicutes in the DSS-treated mice. To deeply explore the effect of inosine on the gut microbiota composition in mice with colitis, PCA, PLS-DA, and SPLS-DA were used to analyze the overall microbial compositions of the mice in the four groups (Figure 6B–D). The results display an obvious separation between the NC and DSS groups, indicating that the DSS treatment significantly shifted the overall microbial composition. However, the inosine intervention obviously recovered the overall microbial composition to a certain extent.

**Figure 7:**
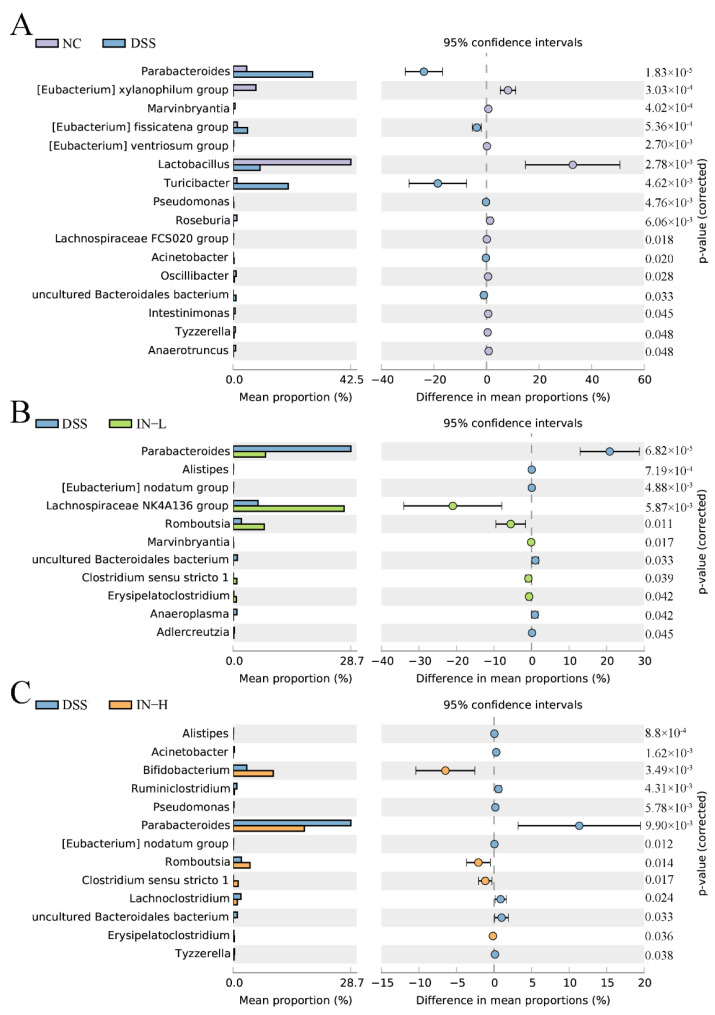
Phylotypes between the NC and DSS groups (**A**), the DSS and IN-L groups (**B**), and the DSS and IN-H groups (**C**) at the genus level were significantly different.

To further identify the alteration of specific microbes at the genus level with the DSS treatment, an extended error bar plot was carried out. As shown in Figure 7A, the disorder of the gut microbiota of the mice in the DSS group was manifested as higher abundances of *Parabacteroides*, (*Eubacterium*) *fissicatena group*, *Turicibacter*, *Pseudomonas*, *Acinetobacter*, and *uncultured Bacteroidales bacterium*, and lower abundances of (*Eubacterium*) *xylanophilum group*, *Marvinbryantia*, *(Eubacterium) ventriosum group*, *Lactobacillus*, *Roseburia*, *Lachnospiraceae FCS020 group*, *Oscilibacter*, *Intestinimonas*, *Tyzzerella*, and *Anaerotruncus*. However, a low dose of inosine intervention significantly elevated the relative abundances of *Lachnospiraceae NK4A136 group*, *Romboutsia*, *Marvinbryantia*, *Clostridium sensu stricto* 1, and *Erysipelatoclostridium*, and significantly decreased the relative abundances of *Parabacteroides*, *Alistipes*, (*Eubacterium*) *nodatum group*, *uncultured Bacteroidales bacterium*, *Anaeroplasma*, and *Adlercreutzia*. Interestingly, a high dose of inosine intervention significantly increased the relative abundances of *Bifidobacterium*, *Romboutsia*, *Clostridium sensu stricto* 1, and *Erysipelatoclostridium*, and significantly decreased the relative abundances of *Alistipes*, *Acinetobacter*, *Ruminiclostridium*, *Pseudomonas*, *Parabacteroides*, (*Eubacterium*) *nodatum group*, *Lachnoclostridium*, *uncultured Bacteroidales bacterium*, and *Tyzzerella*. Together, these data indicate that the inosine intervention was able to recover the structure of the gut microbiota in mice with colitis.

### 2.8. The Associations between Colitis-Related Parameters and Key Microbiota 

**Figure 8:**
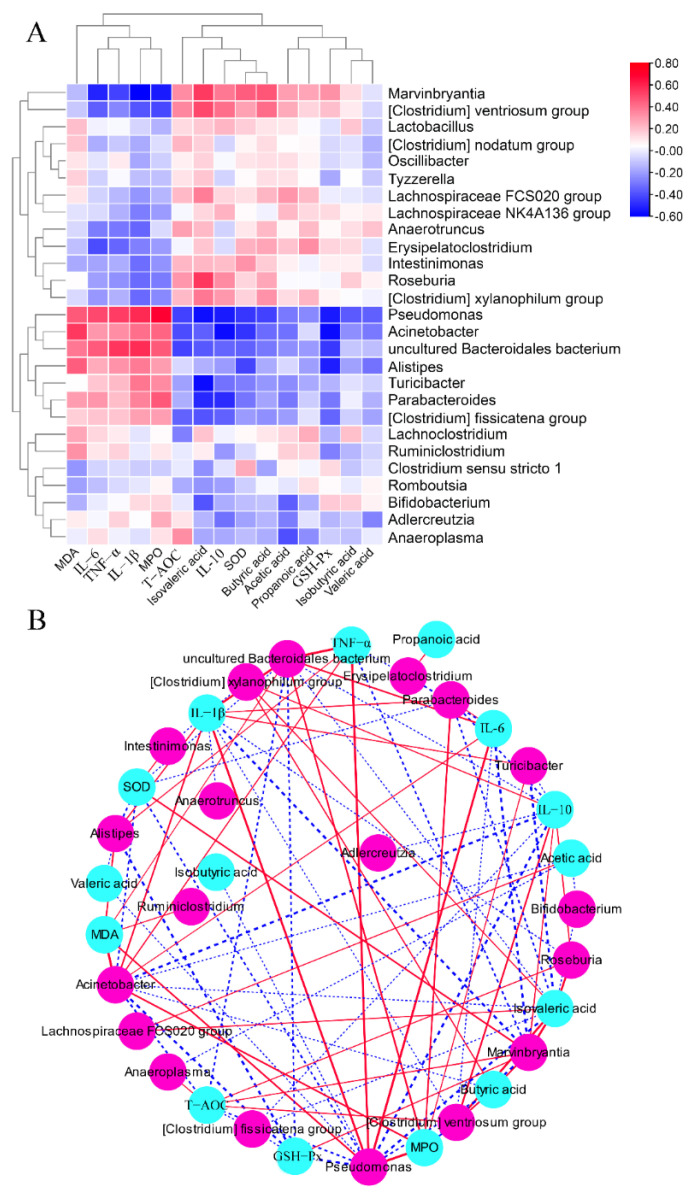
Correlation analysis between the gut microbial genus and some parameters related to colitis. Heatmap (**A**) and network (**B**) of Spearman correlation analysis.

As shown in Figure 8, the concentrations of MDA, IL-6, TNF-α, IL-1β, and MPO were positively associated with the relative abundances of *Pseudomonas*, *Acinetobacter*, *uncultured Bacteroidales bacteriu*, *Alistipes*, *Turicibacter*, *Parabacteroides*, and (*Clostridium*) *fissicatena group*, but were negatively associated with the relative abundances of *Marvinbryantia*, (*Clostridium*) *ventriosum group*, *Lactobacillus*, (*Clostridium*) *nodatum group*, *Oscillibacter*, *Tyzzerella*, *Lachnospiraceae FCS020 group*, *Lachnospiraceae NK4A136 group*, *Anaerotruncus*, *Erysipelatoclostridium*, *Intestinimonas*, *Roseburia*, and (*Clostridium*) *xylanophilum group*. The concentrations of SCFAs and IL-10 and the activities of SOD and GSH-Px were negatively associated with the relative abundances of *Pseudomonas*, *Acinetobacter*, *uncultured Bacteroidales bacteriu*, *Alistipes*, *Turicibacter*, *Parabacteroides*, and (*Clostridium*) *fissicatena group*, while they were positively associated with the relative abundances of *Marvinbryantia*, (*Clostridium*) *ventriosum* group, *Lactobacillus*, (*Clostridium*) *nodatum group*, *Oscillibacter*, *Tyzzerella*, *Lachnospiraceae FCS020 group*, *Lachnospiraceae NK4A136 group*, *Anaerotruncus*, *Erysipelatoclostridium*, *Intestinimonas*, *Roseburia*, and (*Clostridium*) *xylanophilum group*.

## 3. Discussion

The increasing amount of evidence suggests that the occurrence of colitis is associated with heredity, lifestyle, and the environment [15]. The long-term intake of some drugs often causes serious side effects, so it is necessary to explore some active compounds for treating colitis. Inosine is a crucial secondary metabolite in the purine metabolism, which is widely used as a hepatoprotective and anti-inflammatory drug. A previous study exhibited that the oral administration of 800 mg/kg inosine suppressed the differentiation of Th1 and Th2 cells in vitro [15]. We also previously found that 100 mg/kg of inosine prevented LPS-induced acute liver damage [14]. However, the effects of inosine (100 mg/kg or 800 mg/kg) on the intestinal barrier in mice with colitis were not fully investigated. In this study, microbiome-derived inosine intervention effectively prevented DSS-induced colitis by attenuating weight loss and colon shortening, maintaining the intestinal barrier function, and recovering the gut microbiota composition. Particularly, the inosine intervention alleviated the DSS-triggered inflammatory responses and oxidative stress by regulating the NF-κβ and Nrf2 pathways, which can form a basis for comprehensively developing medicinal products related to microbiome-derived metabolites.

### 3.1. Inosine Improved Colonic Inflammatory Responses by Suppressing the NF-κB Pathway

Some pro-inflammatory cytokines can exacerbate the pathogenesis of colitis by regulating intestinal inflammation [16]. In this study, high concentrations of colonic TNF-α, IL-6, and IL-1β were observed in mice with colitis, which was in agreement with a previous study [17]. TNF-α is an essential cytokine mediator in some diseases, whose overproduction enhances proinflammatory and proapoptotic efficacies in various cell types [18]. It was confirmed that TNF-α played a vital role in regulating intestinal epithelial cell apoptosis and survival in the colitis model [19]. In addition, TNF-α was capable of regulating some transcription factors, such as NF-κB, Ets, NF-AT, and AP-1 [18,20]. NF-κB, in particular, was initially considered as a transcription factor to control the expression of the κ Ig in B lymphocytes, which was found to activate the expressions of downstream genes, including iNOS and COX2 [21]. A high expression of iNOS destroys the morphology and promotes the accumulation of nitric oxide. COX2 is an inducible cyclooxygenase that generates an array of downstream lipid mediators, which promotes the development of IBD. In this study, the DSS treatment remarkably promoted the mRNA expressions of colonic NF-κB, iNOS, and COX2, but remarkably suppressed the mRNA expressions of colonic IK-Bα and PPARγ. Interestingly, the inosine intervention significantly alleviated these changes in mice with colitis. PPARγ belongs to the PPARs family, and its expression contributes to suppressing the expression of NF-κB. A previous study exhibited that inosine supplementation can activate the expression of PPARγ by binding to the adenosine A_2A_ receptor, and can help to alleviate inflammatory responses [12]. Additionally, TNF-α has the capability to promote the productions of IL-6 and IL-1β [22]. The accumulation of IL-6 exacerbates local inflammation and destroys organ function by affecting the energy metabolism [23]. IL-1β serves as the vital pro-inflammatory cytokine that mainly stems from activated macrophages and promotes the development of colitis by increasing the intestinal permeability [4]. We found that inosine intervention effectively suppressed DSS-induced increases in the concentrations of colonic TNF-α, IL-6, and IL-1β. These results display that inosine improved the colonic inflammatory responses by suppressing the NF-κB pathway.

### 3.2. Inosine Suppressed the Oxidative Stress by Activating the Nrf2 Pathway

Apart from elevating the anti-inflammation efficacy, the inosine intervention also prevented DSS-induced oxidative stress in mice. Oxygen-derived reactive species stem from the process of cellular metabolism, including the superoxide anion and hydrogen peroxide. Hydrogen peroxide can be converted into hypochlorous acid by the inflammatory enzyme MPO. The overproduction of hypochlorous acid induces oxidative injury to cellular biomolecules, including DNA, lipids, and proteins [5]. In addition, MDA is the genotoxic byproduct during lipid peroxidation and prostaglandin biosynthesis in cells and can affect cell mitosis by destroying the DNA structure [10]. Therefore, MDA and its condensation products are regarded as reliable markers for oxidative stress and are related to many diseases, such as atherosclerosis and IBD [24]. A previous study suggested that inosine can prevent the accumulation of MDA by activating the Nrf2 pathway [25]. The activated expression of Nrf2 promoted the synthesis of antioxidant enzymes (SOD and GSH-Px) that effectively mitigated the development of some diseases by decreasing the accumulation of reactive oxygen species to prevent intestinal cells from oxidative injury. In this study, the inosine intervention dramatically promoted the expression of colonic Nrf2 in mice with colitis. These results exhibit that inosine could suppress oxidative stress by activating the Nrf2 pathway.

### 3.3. Inosine Prevented the Loss of Tight Junction Proteins Induced by DSS

Tight junction proteins have been extensively studied over the past decades because they have a dual role as a barrier and fence to limit the passage of molecules and ions, thereby preventing the occurrence of some inflammatory diseases. The reduction in tight junction proteins decreased the firmness of the intestinal epithelia and elevated the permeability of the intestinal barrier, which elevated the risk of colitis occurrence [26]. Some studies exhibited that DSS treatment reduced the levels of tight junction proteins, including ZO-1, occludin, and claudin-1 [27], among which ZO-1 was the first epithelial tight junction protein identified [28], and showed a direct correlation between the actin and the transmembrane proteins [29]. A previous study exhibited that a deficiency of ZO-1 caused increases in the intestinal barrier permeability and aggravated the severity of colitis [30]. The up-regulated expression of ZO-1 promoted the accumulation of E-cadherin that played an essential role in the junction polarity in both simple and stratified epithelia [31]. In addition, ZO-1 determines the location of the intercellular claudin–claudin polymerization. In the present study, the inosine intervention dramatically up-regulated the expressions of ZO-1, occludin, and claudin-1 in mice with colitis, which partly explained the alleviative efficacy of inosine on the intestinal barrier functions in mice with colitis. Thus, the inosine intervention prevented the loss of tight junction proteins induced by DSS treatment. 

### 3.4. Inosine Induced the Inhibition of Pathogenic Bacteria and the Elevation of Potential Beneficial Bacteria in Mice

Gut microbiota can constitute the intestinal mucosal barrier and is strongly related to the progression of colitis [32]. The disorders of gut microbiota can destroy the microecology of the intestine and further alter the metabolic functions and immune microenvironment, resulting in various intestinal diseases [33]. It was confirmed that the gut microbiota structure was destroyed in UC patients and in mice with colitis [25]. In the present study, the DSS treatment dramatically reduced the relative abundance of Firmicutes and elevated the relative abundance of Proteobacteria, which were in agreement with a previous study [34]. At the genus level, the relative abundances of *Pseudomonas*, *Acinetobacter*, and *Tyzzerella* were increased in mice with colitis. *Pseudomonas* is a Gram-negative pathogen and is strongly related to the development of some inflammatory diseases [35]. *Acinetobacter* is a complex and heterogeneous class of bacteria, and is widely present in patients with intestinal diseases [36]. *Tyzzerella* is a class of commensal human gut bacteria, and its abundance is positively associated with the levels of inflammatory cytokines in IBD patients and mouse models [37]. However, a low dose of inosine intervention dramatically elevated the relative abundances of *Lachnospiraceae NK4A136 group*, *Romboutsia*, *Marvinbryantia*, and *Clostridium sensu stricto* 1 in mice with colitis. *Lachnospiraceae NK4A136 group*, *Romboutsia*, *Marvinbryantia*, and *Clostridium sensu stricto* 1 play essential roles in suppressing the secretion of inflammatory cytokines by producing some beneficial substances, especially SCFAs [38,39,40]. The appropriate concentrations of SCFAs reduced the production of IL-1β by preventing the NLRP3 pathway and regulating the activity of Treg cells by activating GCR43 [41]. In addition, high abundances of *Lachnospiraceae NK4A136 group*, *Romboutsia*, *Marvinbryantia*, and *Clostridium sensu stricto* 1 can suppress the growth of harmful bacteria by competing for intestinal nutrients and spatial substances, which is beneficial for maintaining the intestinal barrier function. Except for *Clostridium sensu stricto* 1, our results also display that *Lachnospiraceae NK4A136 group*, *Romboutsia*, and *Marvinbryantia* were positively associated with the concentrations of cecal SCFAs, and were negatively associated with the level of colonic IL-1β. We speculated that *Clostridium sensu stricto* 1 maintained the intestinal barrier function by secreting extracellular vesicles [42]. Except for *Romboutsia* and *Clostridium sensu stricto* 1, a high dose of inosine intervention dramatically increased the relative abundance of *Bifidobacterium* in mice with colitis. *Bifidobacterium* is a dominant commensal taxon in infant gut microbiota, which is confirmed to promote the production of occludin by combining the Toll-like receptor 2 [43]. These results indicate that the inosine intervention prevented DSS-induced disorders of the gut microbiota in mice, including the inhibition of pathogenic bacteria and the elevation of potential beneficial bacteria.

In summary, microbiome-derived inosine played a significant role in mitigating DSS-induced colitis and offers a potential for therapy in clinical practice. However, more studies are required to reveal the inosine metabolism in the gut and gut microbiota modulation for further mechanistic studies.

## 4. Materials and Methods

### 4.1. Materials

Inosine from *Corynebacterium* spp. was purchased from Sangon Biotech Co., Ltd. (Shanghai, China), and Dextran sodium sulfate (DSS) was purchased from MP Bio (Irvine, CA, USA). Mouse ELISA kits for TNF-α, IL-1β, IL-10, and MPO were obtained from Enzyme-linked Biotech Co., Ltd. (Shanghai, China). Malondialdehyde (MDA), superoxide dismutase (SOD), glutathione peroxidase (GSH-Px), and total antioxidant capacity (T-AOC) were obtained from Jiancheng Bioengineering Co., Ltd. (Nanjing, China). Other reagents used were analytical grade and were obtained from China National Pharmaceutical Group Co., Ltd. (Guangzhou, China).

### 4.2. Animal Experiment Design

Thirty-two male C57BL/6J mice (7 weeks) were obtained from Vital River Laboratory Animal Technology Co., Ltd. (Pinghu, China). The mice were housed in a standard environment (56–65% humidity, 21–24 °C, 12 h light/dark cycle). After seven days of adaptive feeding, the mice were assigned to four groups: normal control (NC), DSS group, low dose of inosine group (IN-L), and high dose of inosine group (IN-H). The mice in the DSS, IN-L, and IN-H groups were provided with distilled water containing 3% DSS (*w*/*v*) from day 7 to day 14. In addition, the mice in the NC and DSS groups were externally gavaged with 0.2 mL of NaCl solution (0.9%, *w*/*v*), while the mice in the IN-L and IN-H groups were externally gavaged with 0.2 mL of inosine solution (100 and 800 mg/kg/day, respectively). At the end of the experiment, the blood was obtained from the orbital plexus and then the mice were sacrificed using cervical dislocation. Protocols for animal experiments were approved by the Ethics Committee of Jiangnan University (JN. no. 20210430c1500608[098]).

### 4.3. Evaluation of the Disease Activity

The disease activity index (DAI) was detected as previously reported [44]. Briefly, the DAI score consisted of three parts, including body weight loss, stool consistency, and fecal blood.

### 4.4. Histopathology Analysis

During tissue harvest, the colons were collected and washed using 1 × PBS, and then fixed with paraformaldehyde (4%, *w*/*v*) at room temperature overnight. The samples were embedded using paraffin, cut into slides of 5 μm in thickness, and stained with hematoxylin and eosin (H&E) for histological analysis. Images were collected using a Panoramic MIDI BF scanner (3D-Histech, Budapest, Hungary). Immunohistochemical staining was carried out according to our previous study [4]. All sections were incubated with the primary antibodies occludin, claudin-1, and ZO-1 at 4 °C for 12 h. Then, the sections were incubated with the secondary antibody (SP9000, ZSGB-Bio, Beijing, China) at 25 °C for 60 min. Panoramic MIDI BF scanner was used to capture images.

### 4.5. Measurement of the Concentrations of Inflammatory Factors

The colon tissues of mice were collected and weighed, and the tissue homogenate (10%, *w*/*v*) was manufactured using pre-chilled PBS. After centrifugation (10,000× *g*, 25 °C, 15 min), the supernatants were collected. The levels of colonic TNF-α, IL-1β, IL-6, and IL-10 were detected using commercial ELISA kits according to the instructions.

### 4.6. Measurement of the Activity of Antioxidant Enzyme

After tissue harvest, the colons were weighed and ground using sterile saline. After centrifugation (10,000× *g*, 25 °C, 15 min), the supernatants were transferred to a new centrifuge tube to determine the levels of MPO and MDA and the activities of SOD, GSH-Px, and T-AOC according to the instructions.

### 4.7. Measurement of Cecal SCFA Concentrations

SCFA concentrations in cecal contents were measured according to our previous study [13]. Briefly, the samples were collected, freeze-dried, weighted, added to 0.5 mL of saturated NaCl solution, and then homogenated. After reaction for 0.5 h, 10 μL of sulfuric acid (10%, *v*/*v*) was transferred to the mixture and then evenly mixed. After centrifugation (10,000× *g*, 4 °C, 15 min), the supernatant was collected and added to 0.8 mL of diethyl ether. The mixture was then centrifugated at 1000× *g* for 15 min at 4 °C to collect the new supernatant. An amount of 0.20 g of sodium sulfate was added to the supernatant and incubated at 25 °C for 0.5 h. After centrifugation at 10,000× *g* for 15 min at 4 °C, the SCFA concentrations in the supernatant were measured using an Agilent 7890B gas chromatographer (Thermo Scientific, Grand Island, NY, USA).

### 4.8. Quantitative Real-Time PCR Analysis

RNA was isolated from the colon using a Trizol reagent (Thermo Scientific, Grand Island, NY, USA), and its integrity was measured via non-denaturing agarose gel electrophoresis. The concentration of total RNA was determined using a nucleic acid quantizer (Implen, MUC, Germany). RNA was reversely translated to complementary DNA using cDNA Synthesis Kit (TAKALA, Dalian, China), and then the relative expression of mRNA levels was determined using a CFX96 Touch™ Real-Time PCR Detection System (Bio-Rad Laboratories, Hercules, CA, USA). The primer sequences in this study are presented in Table S1.

### 4.9. High-Throughput Sequencing

Bacterial DNA was extracted from the fecal samples of each mouse using a commercial kit following the manufacturer’s instructions (MoBio, Carlsbad, CA, USA). The specific region (V3–V4) of the 16S rRNA gene was PCR amplified with universal primers (338F and 806R). The PCR products were verified via 2% agarose gel electrophoresis and the target fragment was cut, collected, and recovered using a QuantiFluor™-ST fluorometer (Promega, Madison, WI, USA). The concentration of target PCR fragment was determined using a NanoDrop Spectrophotometer (Thermo Scientific). The library was established, and high-throughput sequencing was performed on an Illumina HiSeq PE250 platform by Tianhao Biotechnology Company, Ltd. (Shanghai, China).

The original data were treated via QIIME (v. 2.0.0), and the resulting sequences with more than 97% similarity were clustered into the same operational taxonomic unit (OTU). Principal component analysis (PCA), partial least squares discriminant analysis (PLS-DA), and sparse partial least-squares regression discriminant analysis (SPLS-DA) of gut microbiota at the genus were implemented in MetaboAnalyst 5.0. Taxonomic changes that differed significantly between the NC and DSS groups, DSS and IN-L groups, and DSS and IN-H groups were carried out using STAMP (v 2.1.3). The correlations of differed gut microbial phylotypes and biochemical indices were computed using R (v 4.1.3) and presented using Cytoscape (v 3.9.0).

### 4.10. Statistical Analysis

The results were presented as means ± standard deviations. SPSS21 (IBM, Armonk, NY, USA) was applied for one-way analysis of variance. Two-tailed Student’s *t*-test was applied for the analysis of variance between two groups.

## 5. Conclusions

The ameliorative effects of inosine on DSS-induced colitis were investigated, and we found that the inosine intervention suppressed the excretion of inflammatory cytokines by regulating the NF-κB pathway, and elevated the activities of anti-oxidative enzymes by regulating the Nrf2 pathway. In addition, the inosine intervention effectively attenuated colitis by maintaining the intestinal barrier functions and regulating the gut microbiota. This study provides a collective contribution to the understanding of inosine’s potential as a promising method for mitigating intestinal barrier injury.

## Figures and Tables

**Table 1:** The effects of microbiome-derived inosine intervention on the concentrations of cecal SCFAs in DSS-treated mice. * *p* < 0.05 and ** *p* < 0.01 compared with the DSS group.

Groups	SCFAs	Acetic Acid	Propanoic Acid	Isobutyric Acid	Butyric Acid	Valeric Acid	Isovaleric Acid
NC	147.23 ± 32.32 *	105.78 ± 11.89 *	27.35 ± 3.83	3.81 ± 0.38 *	3.72 ± 0.51 *	2.30 ± 0.77	4.26 ± 0.67 **
DSS	118.21 ± 15.43	84.05 ± 15.93	24.00 ± 2.77	3.09 ± 0.45	2.42 ± 0.47	2.27 ± 0.50	2.37 ± 0.56
IN-L	153.32 ± 22.62 *	110.49 ± 22.38 *	29.03 ± 4.31	3.94 ± 1.01	3.64 ± 0.87	2.74 ± 0.75	3.09 ± 0.60 *
IN-H	122.62 ± 42.85	83.34 ± 34.45	25.89 ± 9.07	3.56 ± 1.59	4.34 ± 2.48	2.89 ± 1.79	2.60 ± 1.16 *

## Data Availability

The data shown in the present research are available upon request from the corresponding authors.

## Supplementary Materials

The supporting information can be downloaded at https://www.mdpi.com/article/10.3390/ijms241813852/s1.
