# Supplementary material for: Mitigation of Dextran-Sodium-Sulfate-Induced Colitis in Mice through Oral Administration of Microbiome-Derived Inosine and Its Underlying Mechanisms
Source: Int J Mol Sci. 2023 Sep 8;24(18):13852. doi: 10.3390/ijms241813852 (PMC10530753; doi:10.3390/ijms241813852)
Supplement: Supplementary file 1 [file ijms-24-13852-s001.zip › ijms-2571133-supplementary.pdf]

Supplementary materials

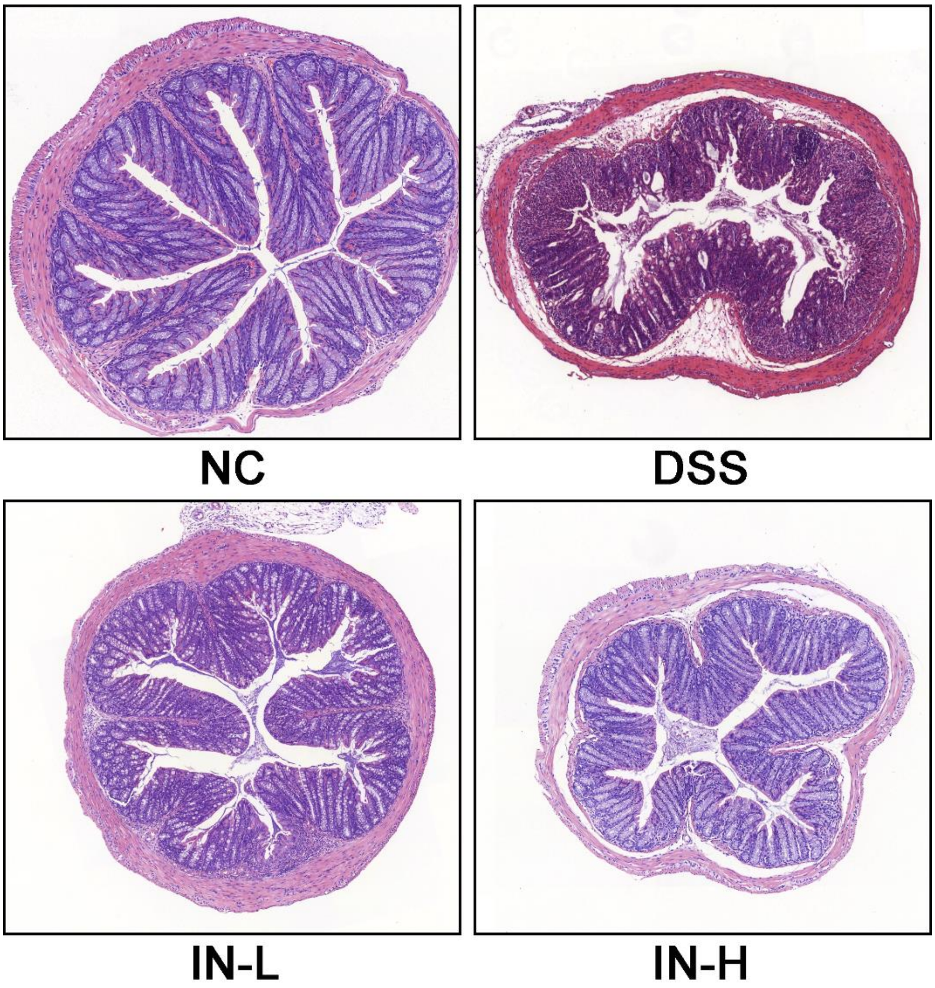

Figure S1. Effects of microbiome-derived inosine intervention on tissue damage in DSS-treated mice.

Table S1. The sequences of RT-qPCR primers.

Primers	Forward (5'-3')	Reverse (5'-3')
ZO-1	CTTCTCTTGCTGGCCCTAAAC	TGGCTTCACTTGAGGTTTCTG
Occludin	CACACTTGCTTGGGACAGAG	TAGCCATAGCCTCCATAGCC
Claudin-1	GATGTGGATGGCTGTCATTG	CCTGGCCAAATTCATACCTG
NF-κβ	CGCCCCTTATCGACCACC	CCTTCTCCCAAGAGTCGTCCA
IK-Bα	ACCAACCAGCCAGAAATCG	TCACAGGCAAGGTGTAGAGGG
COX2	GGGAGTCTGGAACATTGTGAA	GCACGTTGATTGTAGGTGGACTGT
iNOS	CTTGGAGCGAGTTGTGGATTGTC	TAGGTGAGGGCTTGGCTGAGTG
Nrf2	CCTCCGCTGCCATCAGTCAGT	TCGGCTGGGACTCGTGTTCA
PPARγ	AACTGCCGGATCCACAAAAA	AACCTGATGGCATTGTGAGACA
Gapdh	AGGTTGTCTCCTGCGACT	TGCTGTAGCCGTATTCATTGTCA
